# Economic evaluation of a Decision Support Tool to guide intensity of mental health care in general practice: the Link-me pragmatic randomised controlled trial

**DOI:** 10.1186/s12875-022-01839-z

**Published:** 2022-09-16

**Authors:** Mary Lou Chatterton, Meredith Harris, Philip Burgess, Susan Fletcher, Matthew J. Spittal, Jan Faller, Victoria J. Palmer, Patty Chondros, Bridget Bassilios, Jane Pirkis, Jane Gunn, Cathrine Mihalopoulos

**Affiliations:** 1 School of Public Health and Preventive Medicine, Monash University, Level 4, 553 St Kilda Road, Melbourne, VIC 3004 Australia; 2 Deakin Health Economics, Deakin University, Institute for Health Transformation, Geelong, Australia; 3 School of Public Health, The University of Queensland, Brisbane, Australia; 4 The ALIVE National Centre for Mental Health Research Translation, The University of Melbourne, Melbourne, Australia; 5 Department of General Practice, Melbourne Medical School, The University of Melbourne, Melbourne, Australia; 6 Melbourne School of Population and Global Health, The University of Melbourne, Melbourne, Australia

**Keywords:** Economic evaluation, Cost-effectiveness, Mental health, Primary Care, Randomised controlled trial, Care navigation

## Abstract

**Background:**

This paper reports on the cost-effectiveness evaluation of Link-me – a digitally supported, systematic approach to triaging care for depression and anxiety in primary care that uses a patient-completed Decision Support Tool (DST).

**Methods:**

The economic evaluation was conducted alongside a parallel, stratified individually randomised controlled trial (RCT) comparing prognosis-matched care to usual care at six- and 12-month follow-up. Twenty-three general practices in three Australian Primary Health Networks recruited 1,671 adults (aged 18 – 75 years), predicted by the DST to have minimal/mild or severe depressive or anxiety symptoms in three months. The minimal/mild prognostic group was referred to low intensity services. Participants screened in the severe prognostic group were offered high intensity care navigation, a model of care coordination. The outcome measures included in this evaluation were health sector costs (including development and delivery of the DST, care navigation and other healthcare services used) and societal costs (health sector costs plus lost productivity), psychological distress [Kessler Psychological Distress Scale (K10)] and quality adjusted life years (QALYs) derived from the EuroQol 5-dimension quality of life questionnaire with Australian general population preference weights applied. Costs were valued in 2018–19 Australian dollars (A$).

**Results:**

Across all participants, the health sector incremental cost-effectiveness ratio (ICER) of Link-me per point decrease in K10 at six months was estimated at $1,082 (95% CI $391 to $6,204) increasing to $2,371 (95% CI $191 to Dominated) at 12 months. From a societal perspective, the ICER was estimated at $1,257/K10 point decrease (95% CI Dominant to Dominated) at six months, decreasing to $1,217 (95% CI Dominant to Dominated) at 12 months. No significant differences in QALYs were detected between trial arms and the intervention was dominated (less effective, more costly) based on the cost/QALY ICER.

**Conclusions:**

The Link-me approach to stepped mental health care would not be considered cost-effective utilising a cost/QALY outcome metric commonly adopted by health technology assessment agencies. Rather, Link-me showed a trend toward cost-effectiveness by providing improvement in mental health symptoms, measured by the K10, at an additional cost.

**Trial registration:**

Australian and New Zealand Clinical Trials Registry, ANZCTRN 12617001333303.

**Supplementary Information:**

The online version contains supplementary material available at 10.1186/s12875-022-01839-z.

## Background

In Australia, common mental health problems are initially diagnosed and treated by general practitioners (GPs) [1]; however, under- and over-treatment is common. Australian mental health reforms have promoted stepped care to improve the efficiency and quality of mental health care. Evidence suggests that stepped care can be effective for the treatment of depressive and anxiety disorders [2, 3]; few studies have explored the value for money credentials of stepped care approaches for the treatment of anxiety and depression in primary care settings [3].

The Link-me approach to stepped care comprises a patient-completed digital Decision Support Tool (DST) to predict the severity of depressive and anxiety symptoms in three months’ time, support individuals to identify their mental health treatment priorities, and deliver prognosis-based treatment recommendations.

In a pragmatic, stratified randomised controlled trial (RCT), the Link-me intervention resulted in greater reductions in psychological distress at six months over usual care[4]. This paper reports on the economic evaluation incorporated in the RCT [5].

## Methods

The Link-me RCT was conducted within 23 general practices in three Australian states (New South Wales, Victoria, Queensland) across metropolitan, outer metropolitan and regional locations in collaboration with three Primary Health Networks (PHNs). PHNs are not-for-profit companies commissioning health services in 31 geographically defined areas throughout Australia [6]. The study protocol and analysis plan for clinical and economic outcomes are described in full elsewhere [4, 5].

Briefly, adults attending a participating general practice for any reason were invited to complete an eligibility screening tool and the DST (Supporting Information, section S1) on a tablet device in the waiting room. Eligible patients (ages 18–75 years; proficient in English; providing a phone number and email address; having a Medicare card; reporting current anxiety or depression symptoms or use of medication for mental health) were classified into three prognostic groups. Participants categorised into the minimal/mild and severe groups were individually randomised to the intervention or control group. Participants classified as minimal/mild and allocated to the intervention group were offered a selection of low intensity treatment options available through local in-person services, online, by telephone or mobile app. The recommended options were viewed on the tablet immediately after completing the DST and a copy was emailed to participants including links to the relevant services. The severe prognostic group allocated to the intervention were offered care navigation, a model of clinical care coordination informed by the principles of collaborative care and motivational interviewing. Participants could receive up to eight contacts with a trained care navigator to develop and implement a structured care plan to address priorities they identified in the DST. Participants could also access additional care package funding, through the participating PHNs, to support service access when cost was a barrier to care. Participants randomised to the control group for both minimal/mild and severe prognostic groups received advice to discuss any mental health concerns with their GP. The moderate group were excluded from the study based on the availability of mental health service offerings appropriate to this level of need [7].

### Costs

A health sector perspective was adopted as the primary perspective for the economic evaluation with a secondary analysis from a partial societal perspective. Health sector costs included the costs of implementing the Link-me intervention and of other health services used by participants during the trial. Partial societal costs included all health sector costs plus the cost of self-reported productivity losses (Supporting Information, Table S1).

A micro-costing approach was used to estimate the cost of the Link-me intervention and included the resources required for screening, the delivery of care navigation and care packages (Supporting Information, Table S2). Although the control group was screened as part of the study, screening costs were not assigned to this group as screening is not reflective of usual care.

Information on health service use was captured through a self-report resource use questionnaire (RUQ) completed by all participants at six and 12-month follow-ups. The RUQ incorporated questions about the number and type of services accessed (namely, GP, psychologist, psychiatrist, emergency department visits, hospital admissions), medication use, time absent from paid and unpaid work, and days working at reduced capacity while at paid work (presenteeism) due to mental health problems. Standard Australian unit costs were applied (Supporting Information, section S3). Participants were also asked for consent for the study team to access their Medicare Benefits Schedule (MBS) and Pharmaceutical Benefits Scheme (PBS) data. This administrative data provides accurate cost data for government funded health care services and medications.

All costs are presented in 2018–19 Australian dollars (A$). Discounting was not applied as the time horizon was 12 months.

### Health outcomes

Self-report outcome measures were administered at baseline, six-month and 12-month follow-ups. The primary outcome measure was the Kessler Psychological Distress (K10) scale [8] at the six-month follow-up. The K10 was used as an outcome measure in economic analyses. Since lower K10 scores indicate less psychological distress (and a negative change score therefore represents a positive outcome), change in K10 was multiplied by -1 to aid interpretation. The Patient Health Questionnaire 9-item version (PHQ-9) [9], Generalised Anxiety Disorder scale (GAD-7) [10] and EuroQol 5-dimension quality of life questionnaire (EQ-5D-5L) [11] were secondary self-report outcome measures. The EQ-5D-5L was used to measure participants’ preference-based health-related quality of life, and Australian general population preference weights were applied to calculate utility values at each time point [12]. Quality-adjusted life years (QALYs) were calculated from the EQ-5D-5L utility values using the area-under-the-curve (AUC) method [13]. QALYs are the preferred outcome metric in Australia, used by health technology assessment agencies such as the Medical Services Advisory Committee, due to their inherent value for money connotations. An implicit threshold of $50,000 per QALY gained has been used for an intervention to be considered cost-effective, and $28,033 per QALY gained is an additional threshold based on contemporary empirical results [14, 15].

### Statistical analysis

Statistical analyses were conducted using Stata 15 (College Station, Texas, USA). Base case analyses were conducted on an intention-to-treat (ITT) basis. All enrolled participants who completed a baseline assessment were included; however, 63% of participants were missing cost data and 60% were missing QALYs over 12-month follow-up. To address this, missing cost and outcome data were generated using 50 imputed datasets. Imputed values were estimated using variables for randomisation group, age, gender, baseline K10, PHQ-9, GAD-7 and stratified by prognostic group.

The mean difference in costs from both health sector and societal perspectives were estimated using generalised linear models (GLM) with the gamma family and log link. The mean difference between the intervention and control groups in the change in K10 scores from baseline to six and 12-month follow-ups were estimated using multiple linear regression. The difference in mean QALYs between trial arms was estimated using a GLM with the Gaussian family and identity link. All regression and GLM models were estimated with and without adjustment for several baseline covariates specified a priori* (*prognostic group and baseline K10 score) [5, 16]. Pre-specified subgroup analyses were conducted across prognostic groups.

Incremental cost-effectiveness ratios (ICERs) were calculated as the difference in mean costs between the intervention and control groups divided by the difference in mean outcome (change in K10 score, QALYs) by study perspective (health sector and societal). A nonparametric bootstrap with 1,000 iterations from the multiple imputed data was undertaken to estimate 95% confidence intervals (95% CI) around each ICER. The resulting iterations were also used to construct cost-effectiveness planes.

A series of sensitivity analyses explored the effects of: analysing complete cases only, including general practice as a covariate, varying the cost of care navigation sessions to account for efficiencies that may be achieved over time; applying the United Kingdom value set for the EQ-5D-5L [17], and using individual level MBS/PBS data to estimate resource use.

## Results

### Randomised trial

24,616 patients were invited to complete the eligibility screening survey, with 1,671 consenting participants in the trial; 830 classified by the DST into the minimal/mild group and 841 into the severe group (Supporting information, Figure S1). Participants across both trial groups were clinically and demographically similar within prognostic groups and overall (Supporting information, Table S3).

### Costs

The average health sector cost per person invited to complete the screening phase was estimated at $7.34. The cost of care navigators was estimated at $1,144 per participant in the severe prognostic group randomised to the intervention (Supporting information, Table S3). Care package funding was provided to 112 participants allocated to care navigation, at an average cost of $669 per care package recipient (Supporting information Table S4).

Intervention group participants reported significantly more use of mental health nurses (9.4% vs 5.4%) and psychologists (44.1% vs 35.5%) than the comparison group (Supporting information Table S5). The intervention group also reported significantly more days off from unpaid work in the 12 months since trial enrolment (50 vs. 39 days; Supporting information Table S6).

When averaged across all participants, health sector costs were significantly higher among those in the intervention than the control group, at six and 12-month follow-up (Table 1). The adjusted mean difference in health sector costs between groups was $24 (95% CI 9 to 44) at six months increasing to $50 (95% CI 10 to 102) at 12 months. Similarly, for the severe prognostic group, the adjusted mean difference was $340 at six-months (95% CI 126 to 644) increasing to $645 (95% CI -152 to 1818) at 12 months.

**Table 1:** Health sector costs, including intervention costs, according to trial arm, in total sample and stratified by prognostic group

	**All participants**	***p* value**	**Minimal/mild**	***p* value**	**Severe**	***p* value**
			**prognostic group**		**prognostic group**	
**Comparison, n**	837		416		421	
**Intervention, n**	834		414		420	
**6 months**						
Mean cost (SD) [1]						
Comparison	$1,247 (4,473)		$304 (1,045)		$2,178 (6,057)	
Intervention	$2,231 (6,716)		$429 (1,150)		$4,006 (8,984)	
**Mean difference, Coef. (95% CI)**						
Primary analysis [2]	$24 (9 to 44)	*p* < 0.0001	$28 (-5 to 79)	0.106	$340 (126 to 644)	*p* < 0.0001
Sensitivity analysis [3]	$20 (6 to 40)	0.002	$20 (-4 to 60)	0.108	$227 (45 to 514)	0.009
Sensitivity analysis [4]	$24 (7 to 46)	0.002	$17 (-2 to 50)	0.092	$320 (94 to 669)	0.002
Sensitivity analysis [5]	$23 (9 to 42)	*p* < 0.0001	$29 (-3 to 78)	0.078	$287 (96 to 554)	0.001
**12 months**						
Mean cost (SD) [1]						
Comparison	$2,787 (9,879)		$640 (1,801)		$4,908 (13,498)	
Intervention	$3,871 (12,178)		$991 (2,448)		$6,710 (16,476)	
**Mean difference, Coef. (95% CI)**						
Primary analysis [2]	$50 (10 to 102)	0.011	$59 (6 to 134)	0.025	$645 (-152 to 1,818)	0.128
Sensitivity analysis [3]	$31 (9 to 64)	0.003	$31 (-15 to 114)	0.108	$366 (86 to 802)	0.005
Sensitivity analysis [4]	$31 (8 to 62)	0.003	$23 (-10 to 82)	0.214	$492 (143 to 1,031)	0.002
Sensitivity analysis [5]	$48 (8 to 99)	0.014	$59 (9 to 131)	0.018	$538 (-219 to 1,651)	0.19
Sensitivity analysis [6]	$1 (-26 to 47)	0.974	-$3 (-39 to 59)	0.911	$242 (-1,261 to 3,673)	0.822

*SD* Standard deviation, *Coef*. Estimated coefficient, *CI* Confidence interval

[1] Estimated using multiple imputation. [2] Mean for intervention arm minus mean for comparison arm estimated using generalized linear models (gamma family, log link) adjusted for baseline K10 (all models) and prognostic group (model with all participants only). Estimated using multiple imputation. [3] Sensitivity analysis using complete cases only using generalized linear models (gamma family, log link) adjusted for baseline K10 (all models) and prognostic group (model with all participants only). [4] Same as [3] but adjusted for general practice. [5] Sensitivity analysis where care navigators spent 50% of time on care navigation. Data was multiply imputed prior to analysis. [6] Sensitivity analysis using participants with Medicare Benefits Schedule and Pharmaceutical Benefits Scheme data adding covariates of sex and holding a health care card. Only includes 466 participants providing consent to this data (*n* = 238 in comparison group, *n* = 228 in Intervention group)

The adjusted mean difference in societal costs was non-significantly higher in the intervention group across all participants at six ($97; 95% CI -64 to 298) and 12 months ($115; 95% CI -203 to 505) (Table 2). The severe prognostic group had a higher mean difference in societal costs at six months ($678, 95% CI -72 to 1,624) decreasing to $344 (95% CI -1,835 to 3,057) at 12 months, but both differences were non-significant.

**Table 2:** Societal costs, including intervention costs, according to trial arm, in total sample and stratified by prognostic group

	**All participants**	***p* value**	**Minimal/mild**	***p* value**	**Severe**	***p* value**
			**prognostic group**		**prognostic group**	
**Comparison, n**	837		416		421	
**Intervention, n**	834		414		420	
**6 months**						
Mean cost (SD) [1]						
Comparison	$5,575 (12,302)		$2,647 (7,373)		$8,469 (14,906)	
Intervention	$6,529 (13,315)		$2,619 (7,111)		$10,383 ($16,354)	
**Mean difference, Coef. (95% CI)**						
Primary analysis [2]	$97 (-64 to 298)	0.261	$55 (-145 to 351)	0.642	$678 (-72 to 1,624)	0.08
Sensitivity analysis [3]	$109 (-64 to 333)	0.241	$78 (-99 to 369)	0.456	$443 (-159 to 1,280)	0.164
Sensitivity analysis [4]	$144 (-73 to 424)	0.213	$77 (-176 to 482)	0.615	$546 (-133 to 1,431)	0.125
Sensitivity analysis [5]	$83 (-84 to 294)	0.358	$53 (-32 to 645)	0.670	$563 (-176 to 1,489)	0.146
**12 months**						
Mean cost (SD) [1]						
Comparison	$11,022 (21,538)		$4,749 (11,190)		$17,221 (26,903)	
Intervention	$11,553 (23,787)		$5,136 (12,390)		$17,878 (28,856)	
**Mean difference, Coef. (95% CI)**						
Primary analysis [2]	$115 (-203 to 505)	0.505	$179 (-162 to 659)	0.344	$344(-1,835 to 3,057)	0.778
Sensitivity analysis [3]	-$9 (-223 to 272)	0.946	-$93 (-234 to 125)	0.345	-$111 (-2,481 to 2,481)	0.937
Sensitivity analysis [4]	$1 (-243 to 320)	0.993	-$91 (-224 to 118)	0.336	-$24 (-2620 to 3,469)	0.998
Sensitivity analysis [5]	$100 (-228 to 502)	0.56	$177 (-159 to 652)	0.342	$105 (-2142 to 2,919)	0.934
Sensitivity analysis [6]	-$259 (-1,188 to 1,184)	0.673	-$115 (-1,029 to 2,012)	0.872	$341 (-4,420 to 7,740)	0.908

*SD* Standard deviation, *Coef.* Estimated coefficient, *CI* Confidence interval

[1] Estimated using multiple imputation. [2] Mean for intervention arm minus mean for comparison arm estimated using generalized linear models (gamma family, log link) adjusted for baseline K10 (all models) and prognostic group (model with all participants only). Estimated using multiple imputation. [3] Sensitivity analysis using complete cases only using generalized linear models (gamma family, log link) adjusted for baseline K10 (all models) and prognostic group (model with all participants only). [4] Same as [3] but adjusted for general practice. [5] Sensitivity analysis where care navigators spent 50% of time on care navigation. Data was multiply imputed prior to analysis. [6] Sensitivity analysis using participants with Medicare Benefits Schedule and Pharmaceutical Benefits Scheme data adding covariates of sex and holding a health care card. Only includes 466 participants providing consent to this data (*n* = 238 in comparison group, *n* = 228 in Intervention group)

### Health outcomes

The difference between the intervention and control groups in mean K10 score reduction at six months was significant across all participants (mean difference -0.88 95% CI -1.66 to -0.11 *p* = 0.03) and for the severe group (mean difference -1.92 95% CI -3.16 to -0.67 *p* = 0.003). At 12 months the differences were non-significant across all participants (mean difference -0.55 95% CI -1.39 to 0.30, *p* = 0.21) and the severe group (mean difference -1.24 95% CI -2.53 to 0.05, *p* = 0.06; Supporting Information, Table S8).

There were no significant differences in EQ-5D-5L utility values (Supporting Information, Table S5) or QALYs (Table 3) between the intervention and control groups, across all participants at either six or 12-month follow up. In subgroup analysis, the minimal/mild prognostic group had a lower average utility for the intervention group at 12 months (mean difference -0.04 95% CI -0.07 to -0.00, *p* = 0.03).

**Table 3:** Quality adjusted life years (QALYs) calculated from EQ-5D-5L preference-based utility values, according to trial arm, in total sample and stratified by prognostic group

**All participants**	***p* value**	**Minimal/mild symptom group**	***p* value**	**Severe symptom group**		***p* value**
**Comparison, n**	837		416		421	
**Intervention, n**	834		414		420	
**Baseline to 6 months**						
Mean QALYs (SD) [1]						
Comparison	0.610 (0.281)		0.776 (0.162)		0.445 (0.275)	
Intervention	0.606 (0.285)		0.776 (0.163)		0.439 (0.282)	
**Mean difference, Coef. (95% CI)**						
Primary analysis [2]	-0.004 (-0.024 to 0.017)	.733	-0.004 (-0.025to 0.018)	.737	-0.002 (-0.036 to 0.033)	.922
Sensitivity analysis [3]	-0.002 (-0.027 to 0.023)	.883	0.000 (-0.026 to 0.025)	.991	-0.002 (-0.044 to 0.040)	.927
Sensitivity analysis [4]	-0.002 (-0.026 to 0.023)	.901	0.001 (-0.025 to 0.026)	.967	-0.002 (-0.044 to 0.040)	.931
Sensitivity analysis [5]	0.004 (-0.010 to 0.017)	.620	-0.001 (-0.016 to 0.013)	.840	0.009 (-0.013 to 0.031)	.407
Sensitivity analysis [6]	-0.002 (-0.018 to 0.013)	.782	0.000 (-0.015 to 0.012)	.900	-0.001 (-0.029 to 0.026)	.926
**Baseline to 12 months**						
Mean QALYs (SD) [1]						
Comparison	0.621 (0.287)		0.779 (0.173)		0.465 (0.287)	
Intervention	0.613 (0.282)		0.772 (0.166)		0.456 (0.294)	
**Mean difference, Coef. (95% CI)**						
Primary analysis [2]	-0.008 (-0.029 to 0.014)	.481	-0.001 (-0.033 to 0.013)	.400	-0.004(-0.041 to 0.033)	.831
Sensitivity analysis [3]	-0.002 (-0.027 to 0.023)	.883	-0.000 (-0.026 to 0.025)	.991	-0.002 (-0.044 to 0.040)	.927
Sensitivity analysis [4]	-0.002 (-0.026 to 0.023)	.901	0.001 (-0.025 to 0.026)	.967	-0.002 (-0.044 to 0.040)	.931
Sensitivity analysis [5]	0.004 (-0.010 to 0.017)	.620	-0.001 (-0.016 to 0.013)	.840	0.009 (-0.013 to 0.031)	.407
Sensitivity analysis [6]	-0.006 (-0.022 to 0.011)	.506	-0.006 (-0.020 to 0.008)	.414	-0.003 (-0.033 to 0.026)	.827

*SD* Standard deviation, *Coef*. Estimated coefficient, *CI* Confidence interval

[1] Estimated using multiple imputation. [2] Mean for intervention arm minus mean for comparison arm estimated using generalized linear models (Gaussian family, identity link) adjusted for baseline K10 (all models) and prognostic group (model with all participants only). Estimated using multiple imputation. [3] Sensitivity analysis using complete cases only using generalized linear models (Gaussian family, identity link) adjusted for baseline K10 (all models) and prognostic group (model with all participants only). [4] Same as [3] but adjusted for general practice. [5] Sensitivity analysis using multiple imputation adjusted for baseline EQ-5D utility value. [6] Sensitivity analysis using utility values calculated with the UK value set for the EQ-5D-5L, estimated using multiple imputation and generalized linear models (Gaussian family, identity link) adjusted for baseline K10 (all models) and prognostic group (model with all participants only)

### Cost-effectiveness

**Fig. 1:**
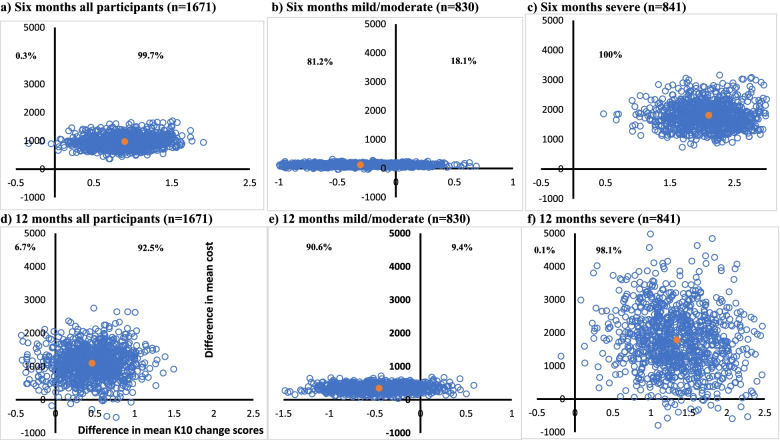
Cost-effectiveness planes for health sector cost per point improvement in K10 score at six and 12 months. **a** Six months all participants (*n* = 1671). **b** Six months mild/moderate (*n* = 830). **c** Six months severe (*n* = 841). **d** 12 months all participants (*n* = 1671). **e** 12 months mild/moderate (*n* = 830). **f** 12 months severe (*n* = 841). Notes: Each cost-effectiveness plane is comprised of 1000 incremental cost-effectiveness ratios (the difference in mean health sector costs divided by the difference in mean K10 scores) between the intervention and control groups estimated through the non-parametric bootstrapping of the multiple imputed data. The percentage of the 1000 cost-effectiveness ratios falling in each quadrant is reported. The upper right-hand quadrant of the plane is where Link-me has both higher incremental costs and benefits (improvement in K10 score) compared to the control group. The upper left-hand quadrant of the plane is where Link-me has higher incremental costs and lower incremental benefits compared to the control group, also referred to as dominated. The lower right-hand quadrant of the plane is where Link-me has lower incremental costs and higher incremental benefits, referred to as dominant. The lower left-hand quadrant of the planes is where Link-me has both lower incremental costs and benefits compared to the control group

**Fig. 2:**
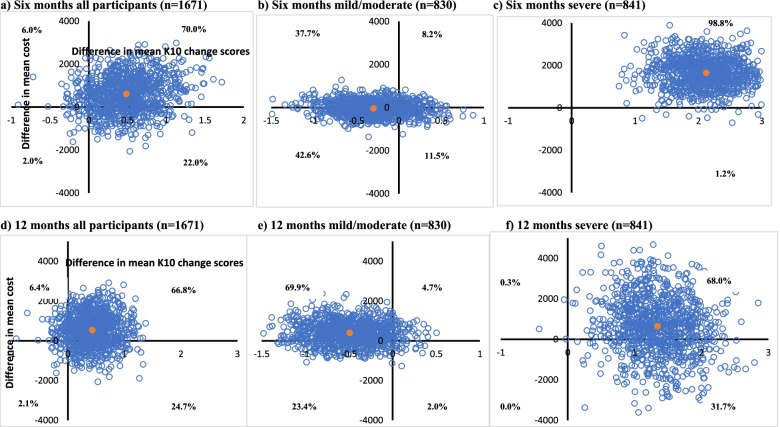
Cost-effectiveness planes for societal cost per point improvement in K10 score at 12 months. **a** Six months all participants (*n* = 1671). **b** Six months mild/moderate (*n* = 830). **c** Six months severe (*n* = 841). **d** 12 months all participants (*n* = 1671). **e** 12 months mild/moderate (*n* = 830). **f** 12 months severe (*n* = 841). Notes: Each cost-effectiveness plane is comprised of 1000 incremental cost-effectiveness ratios (the difference in mean societal costs divided by the difference in mean K10 scores) between the intervention and control group estimated through the non-parametric bootstrapping of the multiple imputed data. The upper right-hand quadrant of the plane is where Link-me has both higher incremental costs and benefits (improvement in K10 score) compared to the control group. The upper left-hand quadrant of the plane is where Link-me has higher incremental costs and lower incremental benefits compared to the control group, also referred to as dominated. The lower right-hand quadrant of the plane is where Link-me has lower incremental costs and higher incremental benefits, referred to as dominant. The lower left-hand quadrant of the planes is where Link-me has both lower incremental costs and benefits compared to the control group

The incremental health sector cost per point decrease in K10 score at six months across all participants was $1,082 (95% CI 391 to 6,204) increasing to $2,371 (95% CI 191 to Dominated) at 12 months (Table 4). From the societal perspective, the cost per point decrease in K10 score at six months was estimated at $1,257 (95% CI Dominant to Dominated), and $1,217 (95% CI Dominant to Dominated) at 12 months. In the minimal/mild prognostic group the cost per point decrease in K10 indicated that the intervention was dominated by the comparison group from both the health sector and societal perspectives. Dominated means that the costs were higher and the mean difference in outcome was negative, in this case the K10 score indicated worse symptoms in the intervention compared to the control group, although the difference was non-significant. Figures 1 and 2 are cost-effectiveness planes providing visual representation of the cost-effectiveness result at six and 12 months.

**Table 4:** Incremental cost-effectiveness ratios using ITT data (imputed) and based on unadjusted cost differences

	**All participants**	**Minimal/mild prognostic group**	**Severe prognostic group**
	***n* = 1671**	***n* = 830**	***n* = 841**
**Health sector perspective**			
ICER (95% CI)			
6 months	$1,082/point improvement on K10	Dominated	$860/point improvement on K10
	(391 to 6,204)	(81 to Dominated)	(366 to 2320)
12 months	$2,371/point improvement on K10	Dominated	$1,326/point improvement on K10
	(191 to Dominated)	(712 to Dominated)	(28 to 8361)
**Societal perspective**			
ICER (95% CI)			
6 months	$1,257/point improvement on K10	$133/point improvement on K10	$776/point improvement on K10
	(Dominant to Dominated)	(Dominant to Dominated)	(82 to 2551)
12 months	$1,217/point improvement on K10	Dominated	$479/point improvement on K10
	(Dominant to Dominated)	(4444 to Dominated)	(Dominant to 8539)

*CI* Confidence interval, *ICER* Incremental cost-effectiveness ratio, *Dominated* Greater costs and less benefit than the comparator, *Dominant* Less costs and greater benefits than the comparator. ICERs and CIs were estimated based on 1,000 bootstrap samples of the multiply imputed data

For the severe prognostic group, the estimated incremental health sector cost per point decrease in K10 score was estimated at $860 (95% CI 366 to 2320) at six months increasing to $1,326 (95% CI 28 to 8361) at 12 months. From the societal perspective the six-month ICER was estimated at $776 per point improved on the K10, decreasing to $479 (95% CI Dominant to 8539) at 12 months.

The lack of a significant difference in QALYs between the intervention and comparison groups from baseline to six-month or 12-month follow-ups, combined with significantly higher health sector costs resulted in Link-me being dominated. Therefore, incremental cost-utility ratios were not calculated.

### Sensitivity analyses

The results were generally robust in sensitivity analyses. Exceptions were the non-significant differences in 12-month health sector costs when using MBS/PBS data and changes to significance in complete case analyses for prognostic sub-groups.

## Discussion

This is the first economic evaluation of a digital platform designed to predict the severity of symptoms and coordinate care for people with depression or anxiety in primary care. Our results suggest that Link-me was associated with additional health sector and societal costs but resulted in achieving additional improvement in mental health symptoms. Incremental costs were highest for the severe prognostic group which also achieved the largest incremental improvement in mental health symptom scores.

Given the time-limited nature of the trial, reported costs may be considered the upper estimate of the true costs associated with delivering care navigation (i.e., training and development) and would likely reduce over time once the intervention became established. The Link‐me intervention was most likely not running at full economic efficiency because the rollout of the actual process of care navigation was being fine‐tuned with the cost of likely learning effects being incurred. If the learning effects were finalised and Link‐me was running at steady state, the throughput of participants would be increased, leading to reductions in average cost per person and contact.

Link-me is a novel approach; therefore, published evaluations of directly comparable interventions are lacking. An economic evaluation of the precursor digital platform to Link-me used to manage the treatment of people with depression found it to be dominant (more effective and less costly) over usual care from both health sector and societal perspectives [18]. It utilised the Assessment of Quality of Life-8D (AQoL-8D) questionnaire [19] to measure utility values and calculate QALYs in contrast to the current evaluation using the EQ-5D-5L. Despite improvements in mental health symptom scores measured with the K10 in the current evaluation, no significant differences were found between groups on the EQ-5D-5L utility scores or QALYs. Economic analyses of collaborative care interventions for treating depression in primary care found mixed results for cost-utility analyses [20, 21]. Studies using the Short Form quality of life measure detected significant differences in QALYs, while those using the EQ-5D found negative or non-significant incremental QALYs [20]. This may reflect lower sensitivity of the EQ-5D to changes in quality of life resulting from improvement in mental health symptoms, despite previous studies indicating that most multi-attribute utility instruments can discriminate between differing levels of symptom severity for people with depression [22]. Link-me provided statistically significant improvement in K10 scores over existing care at six-month follow up, however the mean difference did not reach the prespecified minimal clinically relevant difference of 2.4 points (equivalent to a standardised mean difference of -0.3). However, half of the participants allocated to the Link-me intervention did not accept the offer to engage in care navigation. When a complier average causal effect analysis was undertaken to assess the magnitude of change in K10 scores associated with engagement in the different steps of care navigation, the intervention effect size increased and led to clinically meaningful differences [4]. This suggests that there is an opportunity to optimise the Link-me intervention to improve engagement potentially leading to improved clinical outcomes.

Average health sector and societal costs across all participants for both the Link-me and control groups were higher than reported in previous economic evaluations of stepped and collaborative care interventions for people with depression [3, 20, 21]. The addition of anxiety symptoms to the prognostic model of the DST may have contributed to this result. Previous research found that health sector and productivity costs for comorbid depression and anxiety were significantly higher than either diagnosis alone [23]. Another explanation is that there was increased time missed from work since study participants in both randomised groups were seeking additional treatment for their mental health concerns.

The Link-me intervention was hypothesised to be cost-effective through triaging participants in the minimal/mild prognostic group to low intensity services associated with lower costs and offsetting the increased costs of care in the severe prognostic group. However, participants in the minimal/mild prognostic group who were offered low intensity services had a significantly higher average mean health sector cost at 12‐month follow‐up than their usual care counterparts, in part due to their greater use of psychology services. This may be due to the pragmatic nature of this real-world trial since we could not limit access to more intensive forms of care. Participants in the Link-me group utilised psychological services at a similar rate to the comparison group among the minimal/ mild prognostic group (25% intervention; 19% comparison). The feedback and opportunity for reflection provided by the Link-me DST may have prompted increased help-seeking through psychologists, in preference to the suggested low-intensity options, partially accounting for the higher costs observed.

### Strengths and limitations

The relatively large sample size and embedding of this economic evaluation in a pragmatic RCT engenders confidence in the results. The number and diversity of general practices recruiting study participants suggests that the results are generalisable to similar Australian practices.

This study is also subject to limitations. The use of a self-report measure of health care utilisation and lost productivity are subject to recall bias. However, this would affect both groups in the trial, and previous studies have demonstrated the validity of self-reported RUQs [24]. The sensitivity analyses conducted using accurate administrative data (MBS/PBS) produced results which differed from the primary analyses: namely smaller, non-significant mean differences in health sector costs between the Link-me and control groups. It is important to note that an additional consent was required to access participants’ MBS/PBS data, and only 28% of participants provided this consent, thus limiting the usefulness of this sensitivity analysis.

There was also a high volume of missing data (63.2% control group, 56% Link-me at 12-month follow-up) which may affect the validity of the results. Efforts were made in the design and conduct of the trial to minimise the loss of data, and the analytic approach followed published recommendations for managing missing data [25, 26]. The additional sensitivity analysis to evaluate the effect of imputed missing data did not alter the interpretation of the findings. However, it is unclear if the results would change if all participants completed every follow-up assessment.

## Conclusions

The Link-me approach to stepped mental health care would not be considered cost-effective utilising the cost/QALY outcome metric commonly used by health technology assessment agencies. Link-me could be viewed as trending toward cost-effectiveness by providing improvement in mental health symptoms, assessed by the K10, for additional cost. Given the Productivity Commission’s recommendation for a national digital mental health platform to support a person-centred approach to mental health care in Australia [27], Link-me should be appraised for this important function.

## Supplementary Information

**Additional file 1. **

## Data Availability

Reasonable requests for de-identified data and analytical code will be considered and should be submitted to j.gunn@unimelb.edu.au. Release of data will be subject to approval by relevant ethics and/or monitoring committees and completion of data access agreement form(s).

## Abbreviations

A$: Australian dollars
CI: Confidence Interval
GLM: Generalised linear model
ICER: Incremental cost effectiveness ratio
ITT: Intention-to-treat
MBS: Medicare Benefits Schedule
PBS: Pharmaceutical Benefits Scheme
QALYs: Quality Adjusted Life Years
RCT: Randomised controlled trial
RUQ: Resource use questionnaire
