# Supplementary material for: Economic evaluation of a Decision Support Tool to guide intensity of mental health care in general practice: the Link-me pragmatic randomised controlled trial
Source: BMC Prim Care. 2022 Sep 16;23:236. doi: 10.1186/s12875-022-01839-z (PMC9479277; doi:10.1186/s12875-022-01839-z)
Supplement: Supplementary file 1 — Additional file 1. [file 12875_2022_1839_MOESM1_ESM.docx]

**Economic evaluation of a Decision Support Tool to guide intensity of mental health care in general practice: the Link-me pragmatic randomised controlled trial**

**Supporting Information**

**Section S1.** Decision Support Tool (DST) details

The Link-me DST is a prognostic triage tool designed to be completed by adults in the general practice setting, using a tablet device. Its presentation was developed through an iterative development process employing user-centred design principles to ensure the information is presented in a way that is meaningful and engaging.^1^

The Link-me DST builds on the previously developed *diamond* clinical prediction tool which predicts the severity of depressive symptoms at three months.^2, 3^ Adaptations to the diamond tool were made following advice from an Expert Advisory Panel that it include the prediction of anxiety symptoms as well as depression. By predicting the severity of both these common mental health conditions, the Link-me DST addresses the majority of mental health presentations in the Australian population.^4^ The tool is designed to adopt a biopsychosocial approach to prognosis and comprises 23 items assessing current depressive symptoms, current anxiety symptoms, lifetime history of depression, gender, living situation, ability to manage on available income, self-rated general health, and presence of chronic illness that affects the ability to carry out daily activities.

Two prognostic models embedded within the Link-me DST use an individual’s responses to these items to predict symptom scores for anxiety and depression at three months. Based on their predicted score, individuals are classified into one of three symptom severity groups (minimal/mild, moderate, and severe). Given that an individual may be classified into different severity groups for anxiety and depression, a hierarchy was developed for the combined group which favours the more severe of the two, as depicted in Table 1.

Our preliminary modelling using data from our *diamond* and Target-D studies^2, 3^ suggested that overall, approximately 65 percent of people would be stratified into the minimal/mild symptom severity group, 15 percent into the moderate group, and 20 percent into the severe group.

**Table 1.** Hierarchy of Link-me prognostic group classification

|  |  |  | **Predicted anxiety symptoms** | | |
| --- | --- | --- | --- | --- | --- |
|  |  | **Not eligible^1^** | **Minimal/Mild** | **Moderate** | **Severe** |
|  | **Not eligible^2^** | Not eligible | Minimal/mild | Moderate | Severe |
| **Predicted depressive symptoms** | **Minimal/Mild** | Minimal/mild | Minimal/mild | Moderate | Severe |
|  | **Moderate** | Moderate | Moderate | Moderate | Severe |
|  | **Severe** | Severe | Severe | Severe | Severe |

**Notes:** [1] Not eligible if the sum of the GAD-2 (first two items of the GAD-7^5^) is less than 2. These participants were coded as “**Not eligible for anxiety**”. [2] Not eligible if the sum the PHQ-2^6^ is less than. These participants are coded as “**Not eligible for depressive symptoms**”.

**Table S1.** Impact Inventory as recommended by the Second Panel on Cost-Effectiveness in Health and Medicine

| Sector | Type of Impact | Included in this reference case analysis from …perspective? | | Notes on sources of evidence |
| --- | --- | --- | --- | --- |
|  |  | Health Care Sector | Societal |  |
| Formal Health Care Sector | | | | |
| Health | Health outcomes (effects) | | | |
|  | Longevity effects |  |  |  |
|  | Health -related quality-of-life effects | ✓ | ✓ | EQ-5D-5L |
|  | Other health effects (eg, adverse events and secondary transmissions of infections) | ✓ | ✓ | K10 scores |
|  | Medical costs | | | |
|  | Paid for by third-party payers | ✓ | ✓ | Medications, consultations, hospital care reimbursed by government |
|  | Paid for by patients out-of-pocket | ✓ | ✓ | Consultations, self-help services paid by participant |
|  | Future related medical costs (payers and patients) |  |  |  |
|  | Future unrelated medical costs (payers and patients) |  |  |  |
| Informal Health Care Sector | | | | |
| Health | Patient-time costs | NA |  |  |
|  | Unpaid caregiver-time costs | NA |  |  |
|  | Transportation costs | NA |  |  |
| Non-Health Care Sectors (with examples of possible items) | | | | |
| Productivity | Labour market earnings lost | NA | ✓ | Self- reported paid work loss |
|  | Cost of unpaid lost productivity due to illness | NA | ✓ | Self-reported unpaid work loss |
|  | cost of uncompensated household production | NA |  |  |
| Consumption | Future consumption unrelated to health | NA |  |  |
| Social Services | Cost of social services as part of intervention | NA |  |  |
| Legal or criminal justice | Number of crimes related to intervention | NA |  |  |
|  | Cost of crimes related to intervention | NA |  |  |
| Education | Impact of intervention on educational achievement of population | NA |  |  |
| Housing | Cost of intervention on home improvements (eg, removing lead paint) | NA |  |  |
| Environment | production of toxic waste pollution by intervention | NA |  |  |
| Other (specify) |  | NA |  |  |

Template based on Figure 1 from Sanders et al, JAMA 2016.

**Table S2.** Intervention costing

| Item | Unit cost | Unit | Quantity | Total cost | Reference/Assumptions |
| --- | --- | --- | --- | --- | --- |
| **Screening phase (all participants)** |  |  |  |  |  |
| Survey/DST development |  |  |  | $61,423 |  |
| Survey/DST implementation and maintenance |  |  |  | $153,000 |  |
| iPads to undertake survey/DST | $460.75 | Each | 39 | $17,969 | Assumes outright purchase; no maintenance and working condition at end of 1 year with 5% discount rate on resale price |
| Receptionist time in waiting room [1] | $23.39 | Hours | 410 | $9,595 | 1 minute per encounter; includes 25% on-costs |
| Subtotal without sunk costs |  |  |  | $180,564 |  |
| Subtotal with sunk costs |  |  |  | $241,987 |  |
| **Average health sector cost/person invited to trial without sunk costs** |  |  | 24,616 | **$7.34** | Applied to all participants in intervention arm |
| **Care navigation (severe symptom group only)** |  |  |  |  |  |
| Care navigator training - catering (one day session) | $15.00 | Per person | 11 | $165 | Training 10 care navigators plus the trainer |
| Care navigator training - trainer cost (clinical psychologist) | $1,277.64 | Day | 3 | $3,833 |  |
| Care navigator time (intervention efficiency as experienced in trial) | $49.34 | Hours | 9,653 | $476,280 | 61% of care navigator time spent in clinical activities; includes 25% on-costs |
| Care navigator time (sensitivity analysis) | $49.34 | Hours | 7,912 | $390,393 | Assumed 50% of care navigator time spent in clinical activities; includes 25% on-costs |
| Total care navigation cost as implemented in trial |  |  |  | $480,278 |  |
| Average health sector cost/participant |  |  | 420 | **$1,144** | Base case using average within trial efficiency |
| Total care navigation cost for sensitivity analysis |  |  |  | $394,391 |  |
| Average health sector cost/participant |  |  | 420 | **$939** | Sensitivity analysis assuming greater efficiency |

**Notes**: DST = Decision support tool. [1] Hourly wage estimated using ABS Employee Earnings and Hours, Australia, May 2018; ANZSCO code 5421 Receptionist.

**Section S3.** Costing other resource use

The resource use questionnaire completed as part of the 6-month survey asked participants to report the number of health professional visits (e.g., GP, psychologists, etc), acute care services (e.g., hospitalisations, emergency department visits, ambulance trips), residential care, self-help materials (e.g., online programs, apps and books) and medications to manage their mental health. Health professional visits were costed based on the location of the visit. For visits at a doctor’s room or private practice, a weighted average cost paid by the government for the corresponding health professional, derived from the MBS item reports was used.^7^ Services that occurred in other settings (e.g., community health clinics, hospital outpatient clinics, etc.) were costed using the National Hospital Cost Data Collection Tier 2, non-admitted service event costs.

Since a standard co-payment for health professional visits is not in place under the Medicare Benefits Schedule (MBS), participants were asked to report estimated out of pocket costs paid for these services. Participants also reported the cost of self-help resources which were included in out of pocket costs.

Pharmaceutical Benefits Scheme (PBS) item prices were used to calculate the government and patient out of pocket costs for covered medications.^8^ Online Australian retail pharmacy sites were accessed to determine patient costs for other medications and supplements not covered by the PBS (i.e., Chemist Warehouse, MyChemist, Pharmacy online, Pharmacy direct).

Hospital stays were costed using an average cost for mental health admissions reported by the Australian Institute of Health and Welfare (AIHW). The cost of an ambulance call was based on a national average cost.^9^ Emergency department services were costed using a national average cost from the National Hospital Cost Data Collection. The cost of a day in a residential care unit was estimated from an AIHW mental health services report.

Costs were obtained for the 2018/2019 financial year where possible. If unit costs were reported for previous years, they were inflated to 2018/2019 costs using the AIHW total health price index.

Where explicitly documented, sources of double counting were managed prior to costing. An example would be where a participant reported services in the RUQ that were also accounted for through care packages, such as sessions with a psychologist. The number of sessions paid through a care package would be subtracted from the total number of sessions reported in the RUQ before a unit cost was applied. If the care package funded more sessions than documented in the RUQ, it was assumed that all sessions were funded through the care package and the RUQ sessions were adjusted to zero.

**Costing lost productivity**

The human capital approach was used to value lost paid productivity using an average hourly wage rate calculated from the average weekly earnings reported by the Australian Bureau of Statistics plus 25% overhead costs.^10^ Time off from unpaid activities (i.e., housework) was valued at 25% of the average wage rate plus overhead costs to represent the value of participants’ lost leisure time.^11^

Presenteeism was valued by first calculating the reduced work capacity by subtracting the numeric response regarding the amount of normal work capacity achieved on the days affected by mental health problems from 10 (full work capacity). That result was then divided by 10 to provide a decimal value representing the percentage of time lost in a day; this figure was then multiplied the number of days reported working but bothered by mental health problems and further multiplied by 7.6 hours (estimated in a full-time workday). The resulting value provided the number of hours lost due to presenteeism which was then valued in Australian dollars using the average wage rate plus overhead costs noted above.

Figure S1. Participant flow through the trial


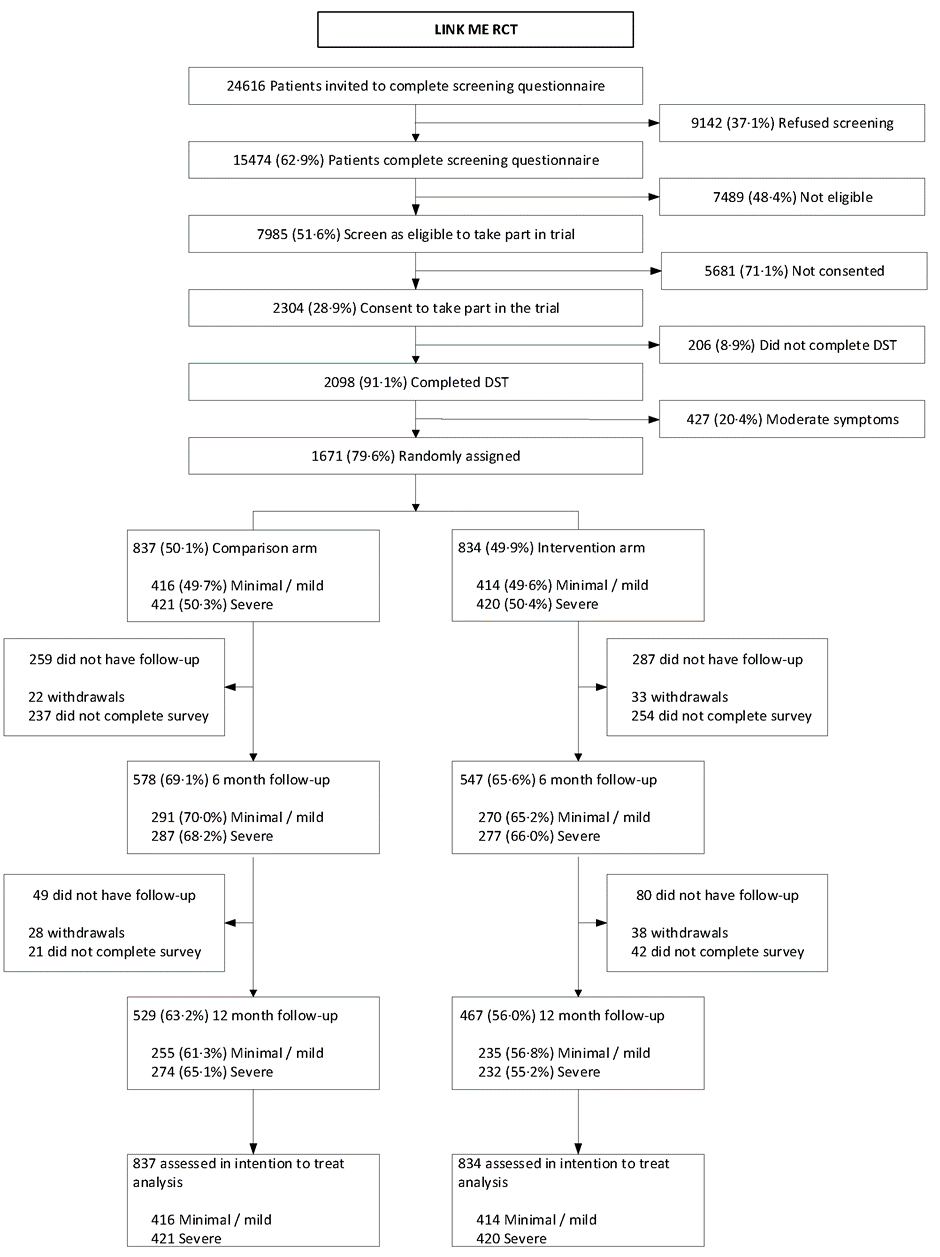


Table S3. Baseline characteristics of Link-me participants according to trial arm, in total sample and stratified by prognostic group (N = 1671)

|  | All participants (comparison) (n=837) | All participants (intervention) (n=834) | Minimal/mild prognostic group (comparison) (n=416) | Minimal/mild prognostic group (intervention) (n=414) | Severe prognostic group (comparison) (n=421) | Severe prognostic group (intervention) (n=420) |
| --- | --- | --- | --- | --- | --- | --- |
|  | Mean (SD) | Mean (SD) | Mean (SD) | Mean (SD) | Mean (SD) | Mean (SD) |
| Age in years | 39.5 (14.8) | 39.7 (15.1) | 40.3 (15.1) | 41.0 (15.5) | 38.7 (14.5) | 38.5 (14.6) |
| Psychological distress (K10) | 24.6 (9.6) | 24.6 (9.7) | 17.3 (4.7) | 16.9 (4.3) | 31.9 (7.5) | 32.2 (7.3) |
| Depressive symptom severity (PHQ-9) | 11.7 (6.7) | 11.7 (6.9) | 5.9 (2.6) | 5.8 (2.4) | 17.4 (4.5) | 17.5 (4.6) |
| Anxiety symptom severity (GAD-7) | 9.4 (5.7) | 9.5 (5.7) | 5.2 (2.8) | 5.2 (2.8) | 13.4 (4.8) | 13.7 (4.5) |
| Overall health (EQ-5D-5L VAS) | 61.2 (22.1) | 58.8 (23.4) | 73.0 (16.7) | 71.7 (18.1) | 49.6 (20.6) | 46.1 (21.0) |
| Quality of life (EQ-5D-5L utility weights) | 0.60 (0.29) | 0.59 (0.30) | 0.78 (0.16) | 0.78 (0.17) | 0.42 (0.28) | 0.40 (0.29) |
|  |  |  |  |  |  |  |
|  | **Median (IQR)** | **Median (IQR)** | **Median (IQR)** | **Median (IQR)** | **Median (IQR)** | **Median (IQR)** |
| Days totally out of role (K10+) | 1 (0 to 5) | 1 (0 to 5) | 0 (0 to 1) | 0 (0 to 1) | 5 (1 to 12) | 5 (1 to 14) |
| Days partially out of role (K10+) | 4 (0 to 12) | 3 (0 to 10) | 1 (0 to 4) | 0 (0 to 4) | 8 (3 to 14.5) | 7 (2 to 14) |
|  | **n (%)** | **n (%)** | **n (%)** | **n (%)** | **n (%)** | **n (%)** |
| Sex |  |  |  |  |  |  |
| **Male** | 236 (28.2) | 221 (26.5) | 119 (28.6) | 106 (25.6) | 117 (27.8) | 115 (27.4) |
| **Female** | 600 (71.7) | 609 (73.0) | 297 (71.4) | 307 (74.2) | 303 (72.0) | 302 (71.9) |
| **Other** | 1 (0.1) | 4 (0.5) | 0 (0.0) | 1 (0.2) | 1 (0.2) | 3 (0.7) |
| Indigenous status |  |  |  |  |  |  |
| **Aboriginal** | 29 (3.5) | 27 (3.2) | 10 (2.4) | 9 (2.2) | 19 (4.5) | 18 (4.3) |
| **Torres Strait Islander** | 0 (0) | 2 (0.2) | 0 (0) | 0 (0) | 0 (0) | 2 (0.5) |
| **Aboriginal and Torres Strait Islander** | 3 (0.4) | 2 (0.2) | 2 (0.5) | 0 (0) | 1 (0.2) | 2 (0.5) |
| **None of the above** | 805 (96.2) | 803 (96.3) | 404 (97.1) | 405 (97.8) | 401 (95.2) | 398 (94.8) |
| Language mainly spoken at home |  |  |  |  |  |  |
| **English** | 812 (97.0) | 797 (95.6) | 397 (95.4) | 387 (93.5) | 415 (98.6) | 410 (97.6) |
| **Other** | 25 (3.0) | 37 (4.4) | 19 (4.6) | 27 (6.5) | 6 (1.4) | 10 (2.4) |
| Highest level of education attained |  |  |  |  |  |  |
| **Below Year 10** | 41 (4.9) | 33 (4.0) | 12 (2.9) | 8 (1.9) | 29 (6.9) | 25 (6.0) |
| **Year 10** | 70 (8.4) | 84 (10.1) | 31 (7.5) | 34 (8.2) | 39 (9.3) | 50 (11.9) |
| **Year 11** | 35 (4.2) | 32 (3.8) | 14 (3.4) | 14 (3.4) | 21 (5.0) | 18 (4.3) |
| **Year 12 or equivalent** | 149 (17.8) | 163 (19.5) | 77 (18.5) | 64 (15.5) | 72 (17.1) | 99 (23.6) |
| **Certificate III/IV** | 182 (21.7) | 150 (18.0) | 80 (19.2) | 78 (18.8) | 102 (24.2) | 72 (17.1) |
| **Advanced diploma / Diploma** | 103 (12.3) | 113 (13.5) | 55 (13.2) | 50 (12.1) | 48 (11.4) | 63 (15.0) |
| **Bachelor degree** | 161 (19.2) | 157 (18.8) | 91 (21.9) | 94 (22.7) | 70 (16.6) | 63 (15.0) |
| **Graduate diploma/Certificate** | 37 (4.4) | 36 (4.3) | 22 (5.3) | 25 (6.0) | 15 (3.6) | 11 (2.6) |
| **Postgraduate degree** | 59 (7.0) | 66 (7.9) | 34 (8.2) | 47 (11.4) | 25 (5.9) | 19 (4.5) |
| Current employment status |  |  |  |  |  |  |
| **Employed** | 574 (68.6) | 522 (62.6) | 332 (79.8) | 299 (72.2) | 242 (57.5) | 223 (53.1) |
| **Unemployed, looking for and available to start work** | 78 (9.3) | 85 (10.2) | 23 (5.5) | 22 (5.3) | 55 (13.1) | 63 (15.0) |
| **Not in labour force** | 185 (22.1) | 227 (27.2) | 61 (14.7) | 93 (22.5) | 124 (29.5) | 134 (31.9) |
| Main activity for those not in labour force |  |  |  |  |  |  |
| **Retired or voluntarily inactive** | 36 (19.5) | 52 (22.9) | 24 (39.3) | 39 (41.9) | 12 (9.7) | 13 (9.7) |
| **Home duties** | 23 (12.4) | 13 (5.7) | 7 (11.5) | 5 (5.4) | 16 (12.9) | 8 (6.0) |
| **Caring for children** | 14 (7.6) | 39 (17.2) | 5 (8.2) | 19 (20.4) | 9 (7.3) | 20 (14.9) |
| **Studying** | 16 (8.6) | 27 (11.9) | 7 (11.5) | 11 (11.8) | 9 (7.3) | 16 (11.9) |
| **Unable to work due to own illness, injury, or disability** | 67 (36.2) | 71 (31.3) | 6 (9.8) | 6 (6.5) | 61 (49.2) | 65 (48.5) |
| **Caring for an ill or disabled person** | 18 (9.7) | 8 (3.5) | 8 (13.1) | 4 (4.3) | 10 (8.1) | 4 (3.0) |
| **Working in an unpaid voluntary job** | 5 (2.7) | 6 (2.6) | 2 (3.3) | 3 (3.2) | 3 (2.4) | 3 (2.2) |
| **Other** | 6 (3.2) | 11 (4.8) | 2 (3.3) | 6 (6.5) | 4 (3.2) | 5 (3.7) |
| Health care card holder | 329 (39.3) | 370 (44.4) | 115 (27.6) | 125 (30.2) | 214 (50.8) | 245 (58.3) |
| Managing on your available income |  |  |  |  |  |  |
| **Easily** | 139 (16.6) | 132 (15.8) | 103 (24.8) | 97 (23.4) | 36 (8.6) | 35 (8.3) |
| **Not too bad** | 309 (36.9) | 299 (35.9) | 197 (47.4) | 192 (46.4) | 112 (26.6) | 107 (25.5) |
| **Difficult some of the time** | 252 (30.1) | 254 (30.5) | 99 (23.8) | 110 (26.6) | 153 (36.3) | 144 (34.3) |
| **Difficult all of the time** | 125 (14.9) | 129 (15.5) | 17 (4.1) | 15 (3.6) | 108 (25.7) | 114 (27.1) |
| **Impossible** | 12 (1.4) | 20 (2.4) | 0 (0.0) | 0 (0.0) | 12 (2.9) | 20 (4.8) |
| Living alone | 134 (16.0) | 129 (15.5) | 44 (10.6) | 45 (10.9) | 90 (21.4) | 84 (20.0) |
| Self-rated health |  |  |  |  |  |  |
| **Excellent** | 38 (4.5) | 39 (4.7) | 28 (6.7) | 29 (7.0) | 10 (2.4) | 10 (2.4) |
| **Very good** | 207 (24.7) | 184 (22.1) | 160 (38.5) | 155 (37.4) | 47 (11.2) | 29 (6.9) |
| **Good** | 302 (36.1) | 321 (38.5) | 175 (42.1) | 183 (44.2) | 127 (30.2) | 138 (32.9) |
| **Fair** | 210 (25.1) | 203 (24.3) | 50 (12.0) | 42 (10.1) | 160 (38.0) | 161 (38.3) |
| **Poor** | 80 (9.6) | 87 (10.4) | 3 (0.7) | 5 (1.2) | 77 (18.3) | 82 (19.5) |
| Long-term illness which limits daily activities | 324 (38.7) | 345 (41.4) | 81 (19.5) | 88 (21.3) | 243 (57.7) | 257 (61.2) |
| Reason for visit to GP |  |  |  |  |  |  |
| **Physical health** | 438 (52.3) | 413 (49.5) | 285 (68.5) | 275 (66.4) | 153 (36.3) | 138 (32.9) |
| **Mental health and wellbeing** | 115 (13.7) | 122 (14.6) | 21 (5.0) | 29 (7.0) | 94 (22.3) | 93 (22.1) |
| **Both physical and mental health** | 206 (24.6) | 212 (25.4) | 57 (13.7) | 50 (12.1) | 149 (35.4) | 162 (38.6) |
| **None of these** | 78 (9.3) | 87 (10.4) | 53 (12.7) | 60 (14.5) | 25 (5.9) | 27 (6.4) |
| History of depression | 522 (62.4) | 514 (61.6) | 130 (31.3) | 143 (34.5) | 392 (93.1) | 371 (88.3) |
| Currently taking medication for mental health | 347 (41.5) | 340 (40.8) | 101 (24.3) | 102 (24.6) | 246 (58.4) | 238 (56.7) |

**Notes:** SD = standard deviation, n = count, IQR = Inter quartile range. Variables contributing to the Link-me DST include: Depressive symptom severity, anxiety symptom severity, gender, managing on your available income, living alone, self-rated health, long-term illness which limits daily activities, and history of depression. Discrepancies in totals due to missing responses.

Table S4. Summary of care package services and use

| Service type | Participants with approved services  n | Participants using the service  n (%) | Total sessions approved  n | Total sessions used  n (%) [1] |
| --- | --- | --- | --- | --- |
| Mental health |  |  |  |  |
| **Psychology** | 48 | 38 (79) | 461 | 213 (46) |
| **Psychiatry** | 15 | 12 (80) | 64 | 43 (67) |
| **Family therapy/counselling** | 1 | 1 (100) | 6 | 6 (100) |
| **Mental health worker** | 1 | 1 (100) | 10 | 10 (100) |
| Allied health |  |  |  |  |
| **Physiotherapy** | 19 | 17 (89) | 163 | 146 (90) |
| **Nutrition/dietary services** | 10 | 8 (80) | 58 | 32 (55) |
| **Exercise physiologist** | 8 | 7 (88) | 108 | 43 (40) |
| **Occupational therapy** | 5 | 4 (80) | 26 | 12 (46) |
| **Chiropractic** | 3 | 3 (100) | 21 | 19 (90) |
| **Osteopathy** | 3 | 2 (67) | 12 | 7 (58) |
| **Podiatry** | 3 | 3 (100) | 7 | 7 (100) |
| **Other allied health professional** | 1 | 1 (100) | 10 | 10 (100) |
| Medical specialists |  |  |  |  |
| **Pain specialist** | 9 | 7 (78) | 26 | 23 (88) |
| **Rheumatology** | 3 | 3 (100) | 4 | 3 (75) |
| **Neurology** | 2 | 2 (100) | 3 | 3 (100) |
| **Gastroenterology** | 1 | 1 (100) | 3 | 3 (100) |
| **Vascular specialist** | 1 | 1 (100) | 2 | 1 (50) |
| **Orthopaedic surgeon** | 1 | 1 (100) | 2 | 1 (50) |
| **Dermatology** | 1 | 1 (100) | 2 | 1 (50) |
| **Metabolic specialist** | 1 | 1 (100) | 1 | 1 (100) |
| Complementary therapies |  |  |  |  |
| **Massage** | 19 | 16 (84) | 114 | 87 (76) |
| **Exercise [2]** | 18 | 12 (67) | 236 | 135 (57) |
| **Yoga** | 13 | 10 (77) | 154 | 106 (69) |
| **Meditation, mindfulness and related training** | 8 | 5 (63) | 28 | 8 (29) |
| **Acupuncture** | 5 | 4 (80) | 44 | 29 (66) |
| **Other [3]** | 2 | 1 (50) | 20 | 10 (50) |
| Support service |  |  |  |  |
| Vocational service | 2 | 1 (50) | 6 | 3 (50) |
| Housing related service | 1 | 1 (100) | 15 | 13.5 (90) |
| Other | 1 | 0 (0) | 1 | 0 (0) |

**Notes**: n = count. Some participants received funding for more than one service. [1] Percentage of sessions paid by PHN compared to the number of sessions approved for funding. [2] Includes Tai Chi, Qi Gong, pilates personal training, group fitness, and water or swimming activities. [3] Includes defence and music classes.

**Table S5.** Participants self-reporting any use of specific services and time off work in the 12 months since trial enrolment, by trial arm and prognostic group (N = 1,071)

| Service type | All participants (comparison) n=549 | All participants (intervention) n=522 |  | Minimal/mild prognostic group (comparison) n=268 | Minimal/mild prognostic group (intervention) n=256 |  | Severe prognostic group (comparison) n=281 | Severe prognostic group (intervention) n=266 |  |
| --- | --- | --- | --- | --- | --- | --- | --- | --- | --- |
|  | **n (%)** | **n (%)** | **p value** | **n (%)** | **n (%)** | **p value** | **n (%)** | **n (%)** | **p value** |
| GP | 317 (57.1) | 334 (62.9) | .05 | 101 (37.4) | 108 (41.5) | .33 | 216 (75.8) | 226 (83.4) | .03 |
| Nurse | 37 (6.7) | 47 (8.9) | .18 | 5 (1.9) | 3 (1.2) | .51 | 32 (11.2) | 44 (16.2) | .09 |
| Mental health nurse | 30 (5.4) | 50 (9.4) | .01 | 2 (0.7) | 1(0.4) | .59 | 28 (9.8) | 49 (18.1) | <.01 |
| Psychiatrist | 103 (18.6) | 121 (22.8) | .09 | 15 (5.6) | 24 (9.2) | .11 | 88 (30.1) | 97 (35.8) | .22 |
| Psychologist | 197 (35.5) | 234 (44.1) | <.01 | 51 (18.9) | 66 (25.4) | .07 | 146 (51.2) | 168 (62.0) | .01 |
| Allied health | 74 (13.3) | 84 (15.8) | .25 | 19 (7.0) | 27 (10.4) | .17 | 55 (19.3) | 57 (21.0) | .61 |
| Other health professional | 36 (6.5) | 40 (7.6) | .49 | 9 (3.3) | 10 (3.8) | .75 | 27 (9.5) | 30 (11.2) | .52 |
| Online therapy | 50 (9.0) | 48 (9.1) | .96 | 17 (6.3) | 17 (6.5) | .91 | 33 (11.6) | 31 (11.6) | 1.0 |
| Smartphone apps | 122 (22.1) | 110 (21.0) | .66 | 44 (16.4) | 43 (16.8) | .91 | 78 (27.5) | 67 (25.0) | .51 |
| Self-help (Books/DVDs) | 149 (27.0) | 150 (28.6) | .55 | 58 (21.6) | 57 (22.3) | .86 | 91 (32.0) | 93 (34.7) | .51 |
| Ambulance | 15 (2.7) | 23 (4.4) | .14 | 0 (0.0) | 3 (1.2) | .08 | 15 (5.3) | 20 (7.5) | .29 |
| Emergency department | 30 (5.4) | 32 (6.1) | .64 | 1 (0.4) | 4 (1.6) | .16 | 29 (10.2) | 28 (10.4) | .93 |
| Time off paid work | 236 (42.8) | 215 (41.0) | .55 | 99 (37.1) | 78 (30.2) | .10 | 137 (48.2) | 137 (51.5) | .44 |
| Time off unpaid work | 215 (39.0) | 215 (41.0) | .50 | 59 (22.1) | 75 (29.1) | .07 | 156 (54.9) | 140 (52.6) | .59 |
| Hospital | 17 (3.1) | 18 (3.4) | .75 | 0 (0.0) | 2 (0.8) | .15 | 17 (6.0) | 16 (6.0) | 1.0 |
| Residential care | 7 (1.3) | 2 (0.4) | .11 | 0 (0.0) | 0 (0.0) | n/a | 7 (2.5) | 2 (0.8) | .11 |
| Medication | 258 (46.6) | 244 (46.2) | .91 | 69 (25.7) | 73 (28.1) | 0.53 | 189 (66.3) | 171 (63.8) | .54 |

**Notes**: Participants were asked about their use of services specifically for their mental health. All p values calculated using Chi-square test

Table S6. Mean number of self-reported visits to services and days off work in the 12 months since trial enrolment, by trial arm and prognostic group

| Service type | All participants  (comparison) | All participants  (intervention) |  | Minimal/mild prognostic group  (comparison) | Minimal/mild prognostic group  (intervention) |  | Severe prognostic group  (comparison) | Severe prognostic group  (intervention) |  |
| --- | --- | --- | --- | --- | --- | --- | --- | --- | --- |
|  | **Mean  (min, max)** | **Mean  (min, max)** | **p value** | **Mean  (min, max)** | **Mean  (min, max)** | **p value** | **Mean  (min, max)** | **Mean  (min, max)** | **p value** |
| GP | 6.36 (1,80) | 5.56 (1,32) | .08 | 3.33 (1,34) | 3.10 (1,27) | .56 | 7.79 (1,80) | 6.74 (1,32) | .11 |
| Nurse | 3.42 (1,24) | 3.66 (1,20) | .74 | 1.60 (1,3) | 1.67 (1,2) | .94 | 3.71 (1,24) | 3.80 (1,20) | .92 |
| Mental health nurse | 5.32 (1,37) | 5.43 (1,49) | .93 | 1.50 (1,2) | 1.00 (1,1) | .72 | 5.62 (1,37) | 5.52 (1,49) | .94 |
| Psychiatrist | 5.04 (1,42) | 4.62 (1,44) | .47 | 2.67 (1,8) | 3.71 (1,18) | .23 | 5.44 (1,42) | 4.85 (1,44) | .39 |
| Psychologist | 7.31 (1,130) | 7.98 (1,70) | .30 | 4.49 (1,23) | 5.30 (1,18) | .24 | 8.31 (1,130) | 9.04 (1,70) | .39 |
| Allied health | 9.70 (1,90) | 7.24 (1, 90) | .09 | 4.47 (1,21) | 4.74 (1,30) | .83 | 11.51 (1,90) | 8.54 (1,90) | .15 |
| Other health professional | 6.61 (1,54) | 6.44 (1,52) | .92 | 8.78 (1,54) | 7.80 (1,52) | .84 | 5.89 (1,52) | 5.97 (1,26) | .96 |
| Ambulance | 1.60 (1,6) | 1.40 (1,3) | .63 | no observations | 1 (1,1) | n/a | 1.60 (1,6) | 1.44 (1,3) | .72 |
| Emergency department | 2.10 (1,10) | 1.75 (1,8) | .38 | 1 (1,1) | 1.25 (1,2) | .84 | 2.14 (1,10) | 1.82 (1,8) | .46 |
| Time off paid work (days) | 41.47 (1,260) | 41.66 (1,260) | .97 | 23.58 (1,260) | 21.19 (1,260) | .58 | 54.41 (2,260) | 53.48 (1,260) | .91 |
| Time off unpaid work (days) | 50.14 (1, 365) | 38.77 (1, 260) | .02 | 21.34 (1,180) | 20.19 (1,110) | .77 | 61.03 (1,365) | 48.59 (2,260) | .08 |
| Hospital (days) | 18.12 (1,120) | 45.64 (1,185) | .77 | no observations | 5.5 (1,10) | n/a | 18.12 (1,120) | 23.07 (1,185) | .64 |
| Residential care | 24.57 (1,120) | no observations | n/a | no observations | no observations | n/a | 24.57 (1,120) | no observations | n/a |

**Notes:** Participants were asked about their use of services specifically for their mental health. All p values calculated using negative binomial regression

**Table S7.** Preference based quality of life outcome as measured by the EQ-5D-5L

|  | **All participants** | **P-value** | **Minimal/mild prognostic group** | **P-value** | **Severe prognostic group** | **P-value** |
| --- | --- | --- | --- | --- | --- | --- |
| **Overall health (EQ-5D-5L VAS)** |  |  |  |  |  |  |
| **Six-month follow-up** |  |  |  |  |  |  |
| Comparison, mean (SD) | 3.94 (24.17) |  | 2.12 (22.33) |  | 5.74 (25.70) |  |
| Intervention, mean (SD) | 5.59 (22.54) |  | 3.76 (19.63) |  | 7.40 (24.93) |  |
| Mean difference, Coef. (95% CI) [1] | 0.11 (-2.43 to 2.65) | 0.93 | 0.78 (-2.40 to 3.96) | 0.63 | -0.53 (-4.44 to 3.38) | 0.79 |
| Mean difference, Coef. (95% CI) [2] | 0.14 (-2.23 to 2.52) | 0.91 | 1.17 (-1.86 to 4.20) | 0.45 | -0.78 (-4.42 to 2.87) | 0.68 |
| Mean difference, Coef. (95% CI) [3] | 0.10 (-2.26 to 2.47) | 0.93 | 1.17 (-1.84 to 4.19) | 0.44 | -0.81 (-4.41 to 2.80) | 0.66 |
| **12-month follow-up** |  |  |  |  |  |  |
| Comparison, mean (SD) | 3.75 (25.79) |  | 0.73 (23.19) |  | 6.73 (27.76) |  |
| Intervention, mean (SD) | 5.99 (24.88) |  | 1.27 (22.70) |  | 10.66 (25.99) |  |
| Mean difference, Coef. (95% CI) [1] | 0.61 (-2.32 to 3.54) | 0.68 | -0.38 (-4.41 to 3.65) | 0.85 | 1.61 (-2.72 to 5.95) | 0.46 |
| Mean difference, Coef. (95% CI) [2] | 0.06 (-2.64 to 2.75) | 0.97 | 0.30 (-3.16 to 3.76) | 0.86 | -0.14 (-4.29 to 4.00) | 0.95 |
| Mean difference, Coef. (95% CI) [3] | -0.07 (-2.74 to 2.59) | 0.96 | 0.30 (-3.13 to 3.74) | 0.86 | -0.32 (-4.33 to 3.69) | 0.88 |
| **Quality of life (EQ-5D-5L utility)** |  |  |  |  |  |  |
| **Six-month follow-up** |  |  |  |  |  |  |
| Comparison, mean (SD) | 0.02 (0.24) |  | -0.00 (0.20) |  | 0.05 (0.28) |  |
| Intervention, mean (SD) | 0.03 (0.25) |  | -0.00 (0.21) |  | 0.07 (0.28) |  |
| Mean difference, Coef. (95% CI) [1] | 0.01 (-0.02 to 0.04) | 0.63 | 0.00 (-0.03 to 0.03) | 0.96 | 0.02 (-0.03 to 0.06) | 0.53 |
| Mean difference, Coef. (95% CI) [2] | 0.00 (-0.02 to 0.03) | 0.91 | -0.00 (-0.03 to 0.03) | 0.86 | 0.01 (-0.03 to 0.05) | 0.72 |
| Mean difference, Coef. (95% CI) [3] | 0.00 (-0.02 to 0.03) | 0.91 | -0.00 (-0.03 to 0.03) | 0.86 | 0.01 (-0.03 to 0.05) | 0.72 |
| **12-month follow-up** |  |  |  |  |  |  |
| Comparison, mean (SD) | 0.04 (0.25) |  | 0.02 (0.20) |  | 0.06 (0.29) |  |
| Intervention, mean (SD) | 0.02 (0.26) |  | -0.02 (0.20) |  | 0.06 (0.30) |  |
| Mean difference, Coef. (95% CI) [1] | -0.02 (-0.05 to 0.01) | 0.17 | -0.04 (-0.07 to -0.00) | 0.03 | -0.01 (-0.06 to 0.05) | 0.79 |
| Mean difference, Coef. (95% CI) [2] | -0.03 (-0.06 to 0.00) | 0.07 | -0.03 (-0.06 to 0.00) | 0.08 | -0.02 (-0.07 to 0.02) | 0.33 |
| Mean difference, Coef. (95% CI) [3] | -0.03 (-0.06 to -0.00) | 0.05 | -0.03 (-0.06 to 0.00) | 0.08 | -0.03 (-0.07 to 0.02) | 0.29 |

**Notes:** SD = Standard Deviation; Coef. = Estimated coefficient; RR = Rate ratio; CI = Confidence Interval. [1] Mean for intervention arm minus mean for comparison arm estimated using linear regression adjusted for baseline outcome measure (all models) and prognostic group (model with all participants only). Estimated using multiple imputation. [2] Sensitivity analysis using complete cases only with linear regression adjusted for baseline outcome measure (all models) and prognostic group (model with all participants only). [3] Same as 2 but adjusted for general practice treated as random intercept. [4] Mean difference from 1 calculated relative to the pooled SD of baseline scores. [5] Rate ratio estimated using negative binomial regression adjusted for baseline days out of role (all models) and prognostic group (model with all participants only). Estimated using multiple imputation. [6] Sensitivity analysis using complete cases only with negative binomial regression adjusted for baseline days out of role (all models) and prognostic group (model with all participants only). [7] Same as 6 but adjusted for general practice treated as random intercept.

Table S8. K10 Psychological distress scores according to trial arm, in total sample and stratified by prognostic group

|  | **All participants** | **P-value** | **Minimal/mild prognostic group** | **P-value** | **Severe prognostic group** | **P-value** |
| --- | --- | --- | --- | --- | --- | --- |
| **Intervention, n** | 837 |  | 416 |  | 421 |  |
| **Comparison, n** | 834 |  | 414 |  | 420 |  |
| **Six-month follow-up** |  |  |  |  |  |  |
| **Mean change, mean (SD) [1]** |  |  |  |  |  |  |
| Comparison | -0.32 (7.40) |  | 2.18 (6.20) |  | -2.78 (7.66) |  |
| Intervention | -1.18 (8.09) |  | 2.50 (5.80) |  | -4.81 (8.37) |  |
| **Mean difference, Coef. (95% CI)** |  |  |  |  |  |  |
| Primary analysis [2] | -0.88 (-1.66 to -0.11) | 0.03 | 0.16 (-0.76 to 1.08) | 0.73 | -1.92 (-3.16 to -0.67) | 0.003 |
| Sensitivity analysis [3] | -0.99 (-1.74 to -0.24) | 0.01 | 0.07 (-0.84 to 0.99) | 0.87 | -2.05 (-3.23 to -0.86) | <0.001 |
| Sensitivity analysis [4] | -0.99 (-1.74 to -0.24) | 0.01 | 0.07 (-0.83 to 0.98) | 0.87 | -2.05 (-3.23 to -0.87) | <0.001 |
| **SMD (95% CI) [9]** | -0.09 (-0.17 to -0.01) | 0.03 | 0.04 (-0.17 to 0.24) | 0.73 | -0.26 (-0.43 to -0.09) | 0.003 |
| **12-month follow-up** |  |  |  |  |  |  |
| **Mean change, mean (SD) [1]** |  |  |  |  |  |  |
| Comparison | -0.88 (7.83) |  | 1.68 (6.15) |  | -3.42 (8.44) |  |
| Intervention | -1.41 (7.85) |  | 2.00 (6.14) |  | -4.77 (7.89) |  |
| **Mean difference, Coef. (95% CI)** |  |  |  |  |  |  |
| Primary analysis [2] | -0.55 (-1.39 to 0.30) | 0.21 | 0.13 (-0.90 to 1.16) | 0.80 | -1.24 (-2.53 to 0.05) | 0.06 |
| Sensitivity analysis [3] | -0.59 (-1.39 to 0.20) | 0.14 | 0.21 (-0.75 to 1.17) | 0.67 | -1.42 (-2.68 to -0.16) | 0.03 |
| Sensitivity analysis [4] | -0.59 (-1.38 to 0.21) | 0.15 | 0.22 (-0.73 to 1.17) | 0.65 | -1.42 (-2.67 to -0.16) | 0.03 |
| **SMD (95% CI) [9]** | -0.06 (-0.14 to 0.03) | 0.21 | 0.03 (-0.20 to 0.26) | 0.80 | -0.17 (-0.34 to 0.01) | 0.06 |

**Notes:** SD = Standard deviation; Coef. = Estimated coefficient; CI = Confidence Interval; SMD = Standardised mean difference. [1] Estimated using multiple imputation. [2] Mean for intervention arm minus mean for comparison arm estimated using linear regression adjusted for baseline outcome measure (all models) and prognostic group (model with all participants only). Estimated using multiple imputation. [3] Sensitivity analysis using complete cases only with linear regression adjusted for baseline outcome measure (all models) and prognostic group (model with all participants only). [4] Same as 3 but adjusted for general practice using a linear mixed effects model with practice as a random intercept. [5] CACE analysis: undertaken in the severe prognostic group only. Conducted using two-stage least squares instrumental variable regression where the adherence variable is a binary coded variable representing participants attended at least one appointment with the care navigator and there was a match between patient priorities and the action plan. Estimated using multiple imputation. [6] CACE analysis: Same as 5 except the adherence variable is a binary coded variable representing (a) the participant had at least one structured contact with the care navigator and there was a match between participant priorities and the treatment plan, and (b) a referral was made to other services. Estimated using multiple imputation. [7] CACE analysis: Same as 5 except the adherence variable is a binary coded variable representing (a) the participant had at least one structured contact with the care navigator and there was a match between participant priorities and the treatment plan, (b) a referral was made to other services, and (c) the participant was approved for care package funding. Estimated using multiple imputation. [8] CACE analysis: Same as 5 except the adherence variable is a binary coded variable representing (a) the participant had at least one structured contact with the care navigator and there was a match between participant priorities and the treatment plan, (b) a referral was made to other services, (c) the participant was approved for care package funding, and (d) some or all of the care package funding was spent. Estimated using multiple imputation. [9] Mean difference in the primary analysis calculated relative to the pooled SD of baseline scores.

**Table S9.** EQ-5D-5L utility values calculated using the UK value set according to trial arm, in total sample and stratified by prognostic group

|  | All participants (comparison) (n=837) | All participants (intervention) (n=834) | Minimal/mild prognostic group (comparison) (n=416) | Minimal/mild prognostic group (intervention) (n=414) | Severe prognostic group (comparison) (n=421) | Severe prognostic group (intervention) (n=420) |
| --- | --- | --- | --- | --- | --- | --- |
|  | Mean (SD) | Mean (SD) | Mean (SD) | Mean (SD) | Mean (SD) | Mean (SD) |
| Baseline | 0.60 (0.29) | 0.59 (0.30) | 0.78 (0.16) | 0.78 (0.17) | 0.42 (0.28) | 0.40 (0.29) |
| Six-month follow-up | 0.62 (0.37) | 0.62 (0.42) | 0.77 (0.23) | 0.78 (0.25) | 0.47 (0.41) | 0.47 (0.46) |
| 12-month follow-up | 0.64 (0.37) | 0.61 (0.40) | 0.79 (0.25) | 0.76 (0.28) | 0.49 (0.39) | 0.46 (0.47) |

**Notes:** SD = Standard deviation, n = count, all values estimated after multiple imputation

**References**

1. Wachtler C, Coe A, Davidson S, Fletcher S, Mendoza A, Sterling L, et al. Development of a mobile clinical prediction tool to estimate future depression severity and guide treatment in primary care: User-centred design. JMIR Mhealth Uhealth. 2018;6(4):e95.

2. Chondros P, Davidson S, Wolfe R, Gilchrist G, Dowrick C, Griffiths F, et al. Development and validation of a prognostic model for predicting depression severity in adult primary patients with depressive symptoms using the diamond longitudinal study. Journal of Affective Disorders. 2018;227:854-60.

3. Gunn J, Wachtler C, Fletcher S, Davidson S, Mihalopoulos C, Palmer V, et al. Target-D: A stratified individually randomized controlled trial of the diamond clinical prediction tool to triage and target treatment for depressive symptoms in general practice: Study protocol for a randomized controlled trial. Trials. 2017;18:342.

4. Australian Bureau of Statistics. National Survey of Mental Health and Wellbeing: Summary of results. Canberra: ABS; 2008.

5. Spitzer RL, Kroenke K, Williams JBW, Lowe B. A brief measure for assessing generalized anxiety disorder: The GAD-7. Arch Intern Med. 2006;166(10):1092-7.

6. Kroenke K, Spitzer RL, Williams JBW. The Patient Health Questionnaire-2: Validity of a two-item depression screener. Med Care. 2003;41(11):1284-92.

7. Australian Government. Medicare Australia Statistics, Medicare Item Reports 2019 [Available from: <http://medicarestatistics.humanservices.gov.au/statistics/mbs_item.jsp>.

8. Australian Government. Medicare Australia Statistics, Pharmaceutical Benefits Schedule Item Reports 2019 [Available from: <http://medicarestatistics.humanservices.gov.au/statistics/pbs_item.jsp>.

9. Australian Government. Report on Government Services: Ambulance services 2019 [Available from: <https://www.pc.gov.au/research/ongoing/report-on-government-services/2019/health/ambulance-services/rogs-2019-parte-chapter11.pdf>.

10. Australian Bureau of Statistics. Average weekly earnings, Australia, Nov 2018 2019 [Available from: <https://www.abs.gov.au/ausstats/abs@.nsf/mf/6302.0>.

11. Jacobs P, Fassbender K. The measurement of indirect costs in the health economics evaluation literature - A review. International Journal of Technology Assessment in Health Care. 1998;14(4):799-808.
